# Peripheral Macrovascular Involvement in Systemic Sclerosis: A Cohort Study by Color and Spectral Doppler Ultrasonography

**DOI:** 10.3390/life13020487

**Published:** 2023-02-10

**Authors:** Roberto D’Alessandro, Estrella Garcia Gonzalez, Paolo Falsetti, Edoardo Conticini, Miriana d’Alessandro, Enrico Selvi, Francesca Bellisai, Virginia Berlengiero, Giulia Vallifuoco, Anna Paola Pata, Marco Bardelli, Caterina Baldi, Luca Cantarini, Elena Bargagli, Bruno Frediani

**Affiliations:** 1 Rheumatology Unit, Department of Medicine, Surgery and Neurosciences, University of Siena, 53100 Siena, Italy; 2 Respiratory Diseases and Lung Transplantation Unit, Department of Medicine, Surgery and Neurosciences, University of Siena, 53100 Siena, Italy

**Keywords:** systemic sclerosis, Raynaud phenomenon, ultrasonography, ulnar artery, ulnar artery occlusion, vasculopathy, resistance index, digital ulcer, fibrosis

## Abstract

Objectives: Systemic sclerosis (SSc) is a disease characterized by diffuse sclerosis of skin and organs and small vessel vasculopathy. Despite it, large vessels can also be involved with ulnar artery vasculopathy, revealing as a more frequent feature of SSc. The aim of this paper is to assess the macrovascular involvement of SSc patients through an ultrasound (US) evaluation of radial and ulnar arteries. Methods: Radial and ulnar resistance indices (RIs) and peak systolic velocity (PV) (cm/s) together with clinical features of SSc patients were evaluated. Raynaud phenomenon (RP) and healthy control (HC) groups were used for comparison. Results: Forty-three SSc patients were evaluated. Twelve patients (28%) had ulnar artery occlusions (UAOs). In nine cases (75%), UAOs were bilateral. A high UAO prevalence (42%) was found in SSc patients with late nailfold-video-capillaroscopy (NVC) pattern (*p* = 0.0264). Patients with UAOs had digital ulcers (DUs) in 10 cases (83.3%). Radial and ulnar PVs were lower in SSc and RP patients than the HC group. Radial and ulnar RIs were higher in SSc and RP patients than the HC group. A decision tree analysis led to the classification of 70% of SSc patients with an ulnar RI > 0.82 and ulnar PV > 2.8 cm/s. The most influential variables on UAO development were interstitial lung disease (ILD) (*p* = 0.002) and NVC pattern (*p* = 0.002). A positive correlation was shown between modified Rodnan skin score (mRSS) and ILD (*p* = 0.283; r = 0.033), mRSS and DU (r = 0.344; *p* = 0.012) and DU and ILD (r = 0.303; *p* = 0.024). Male sex was associated with increased UAO frequency (*p* = 0.042). Conclusions: UAO is a peculiar feature of severe SSc present in 28% of the cases, particularly associated with the presence of ILD and late NVC pattern. In 75% of the cases, UAOs are bilateral. DUs are very frequent in patients with UAOs (83%). The RI evaluated by US could be useful to distinguish SSc from HC patients. US could be a useful tool for assessing high-risk DU development in patients.

## 1. Introduction

Systemic sclerosis (SSc) is a connective tissue disease (CTD) characterized by diffuse cutaneous sclerosis of skin and organs, small vessel vasculopathy and immune system dysregulation associated with the production of autoantibodies [1,2]. In the pathophysiological process, the innate and adaptive immune cells and fibroblast dysregulation lead to the production of pro-inflammatory and pro-fibrosing proteins such as Wnt or TGF-β pathways, finally causing vasculopathy and fibrosis [3]. Although small vessel vasculopathy can be considered a hallmark of this disease, large vessels can also be involved [1]. Accordingly, involvement of medium vessels such as the ulnar artery has been evaluated in the literature, with results showing how ulnar artery occlusion (UAO) could be considered as a severity marker of vasculopathy as well as a predictive marker of digital ulcers (DUs), which are typically more frequent in patients with limited cutaneous (lc) SSc [4,5,6,7]. DUs are one of the most common complications of SSc vasculopathy, representing an important burden for the patients [8,9]. On the other side, Raynaud’s phenomenon (RP), caused by impaired digital perfusion, can occur as a primary phenomenon or secondary to a wide range of underlying causes and is a typical presentation sign of SSc [10]. Furthermore, RP is one of the classification criteria of SSc and an important feature of vasculopathy [11]. It must be pointed out that medium or large vessel involvement has recently been reported as a feature of SSc vasculopathy. Indeed, Bandini et al. showed how potentially also the splanchnic vessels in SSc may be non-invasively investigated with abdominal US and color Doppler US, showing that some morphological and functional US parameters of mesenteric arteries of SSc patients are different from healthy controls defining a “bowel vasculopathy” [12]. In addition, renal arteries seem to be involved in SSc vasculopathy, often sub-clinically, and are characterized by vascular damage and normal renal function and expressed by an increase in intrarenal stiffness [13]. Finally, Hughes et al. have recently described an overlap entity of SSc associated with the presence of antineutrophil cytoplasm autoantibodies (ANCA), which could represent a poor prognostic vascular phenotype [14]. Regarding the ulnar arteries in SSc, a multicenter cross-sectional study evaluated the association of UAO, assessed using Doppler ultrasound (US), with the severity markers of SSc, confirming that UAO may represent a relevant severity indicator of vasculopathy in this disorder. However, a limited number of papers have assessed the different vascular US patterns in patients affected by SSc and RP compared to healthy controls (HC) and primary RP patients [15,16,17]. This methodology demonstrated good accuracy, safety and noninvasiveness, showing a relevant repeatability for properly experienced users. The aim of this paper was to evaluate the macrovascular involvement in a group of SSc patients through a US approach to the study of radial and ulnar arteries compared to a group of RP and HC subjects with a particular attention to the eventual UAO in a Caucasian group of patients.

## 2. Materials and Methods

### 2.1. US Evaluation

The macrovascular involvement was assessed using the resistance index (RI) and peak systolic velocity (cm/s) (PV) of radial and ulnar arteries measured by ultrasonography color Doppler (CDUS) with spectral wave analysis (SWA). One ultrasound-experienced rheumatologist performed US unaware of subjects’ clinical history. Each examination was performed in the same room with a stable temperature (23–25 °C), using a high-frequency probe (10–22 MHz) of “Esaote MyLab Twice”. CDUS was performed at Guyon’s canal of both wrists. RI, defined as [peak systolic velocity—peak diastolic velocity]/peak systolic velocity), and PV of radial and ulnar arteries were measured.

### 2.2. Study Population

Forty-three SSc patients followed at Siena University Hospital were consecutively enrolled from September 2020 to June 2021. Patients fulfilling 2013 ACR/EULAR SSc classification criteria were enrolled [5]. The following data were collected for each patient: age, sex, body mass index (BMI), smoking history, hypertension, disease duration, DU (active DU or previous healed DU), modified Rodnan skin score (mRSS), type of cutaneous involvement (limited/diffuse), serum anticentromere antibodies (ACA), anti PM/Scl antibodies, anti-topoisomerase I antibodies (Scl-70), pulmonary artery pressure (PAPs) expressed in mmHg and left ventricular ejection fraction (FE%) evaluated at echocardiography, diffusion capacity for carbon monoxide (DLCO), nailfold-video-capillaroscopy (NVC) pattern (early, active and late) and macrovascular CDUS parameters (RI and PV). Treatment at the time of the evaluation was reported: antiplatelet therapy, calcium channel blockers, iloprost, endothelin receptor antagonists (ERA), phosphodiesterase inhibitors, immunosuppressive treatment (mycophenolate mofetil, methotrexate, azathioprine, rituximab), nintedanib and steroids. The results from the SSc group were compared to those of a group of patients affected by primary RP (n = 23) and to HCs (n = 22). Data regarding RP and DU severity and diagnosis of pulmonary hypertension made by right heart catheterization were not evaluated. All patients were evaluated under vasodilator treatment except for those receiving iloprost, who were evaluated on a different day with respect to the treatment. All patients gave their own written informed consent. This study was specially approved by the ethical committee of “Azienda Ospedaliero Universitaria Senese” (protocol number: RHELABUS 22271).

## 3. Statistical Analysis

Results are expressed as mean ± standard deviation. For categorical variables, Fisher’s exact or Chi-squared tests were used to compare proportions between groups. Non-parametric one-way ANOVA (Kruskal–Wallis test) and Dunn test were performed for multiple comparisons. Spearman’s test was used to test correlation of clinical parameters. Receiver operating characteristic (ROC) curve analysis was performed to find the best cut-off values of ulnar and radial PVs and RIs to distinguish SSc, RP and HC groups. A classification and regression decision tree was constructed to determine the best clustering variables according to the Gini criterion. We created a series of test/training partitions to evaluate the accuracy of potential binary classifiers by means of a confusion matrix. Supervised Principal Component Analysis (PCA) was used in an explorative approach to identify trends in immunological features by 2D representation of the multi-dimensional data set. To understand or predict the effect of clinical features (patterns in the capillaroscopy, disease duration, treatment, DU and radial and ulnar PV and RI) on UAO (presence versus absence), logistic regression analysis was performed, and the odds ratio (OR), 95% confidence interval (CI), and *p* values were calculated. A *p* value less than 0.05 was considered statistically significant. Statistical analysis was performed by GraphPad Prism 9.4 and XLSTAT 2021 software.

## 4. Results

### 4.1. Study Population Results

Forty-three SSc patients were evaluated. Thirty-seven patients were female (86%) and six were males (14%), and mean age was 60.5 ± 15. Mean body mass index was 24.2 ± 4.2. Seventeen patients (39%) were hypertensive. All patients showed ANA positivity. Twenty patients (46.5%) were ACA+, thirteen were Scl70+ (34.8%), two were PM/Scl+ (4.6%) and eight (18.6%) had no specific autoantibodies. Twenty-eight (65%) had lc SSc and fifteen (35%) had diffuse cutaneous SSc. We summarized in Table 1 the general and clinical characteristics of SSc, RP and HC groups.

### 4.2. CDUS Results

Radial PV was lower in SSc and RP patients than HCs (*p* = 0.0032 and *p* = 0.0135, respectively). On the contrary, the radial RI was higher in SSc and RP patients than HCs (*p* < 0.0001 and *p* = 0.0042, respectively). The ulnar PV was lower in SSc and RP patients than the HC group (*p* < 0.0001 and *p* = 0.0171, respectively). The ulnar RI was higher in SSc and RP than HC (*p* < 0.0001 and *p* = 0.0065, respectively). US results are reported in Table 2.

### 4.3. Decision Tree Model, ROC Curve and Clinical Correlation Results

**Figure 1:**
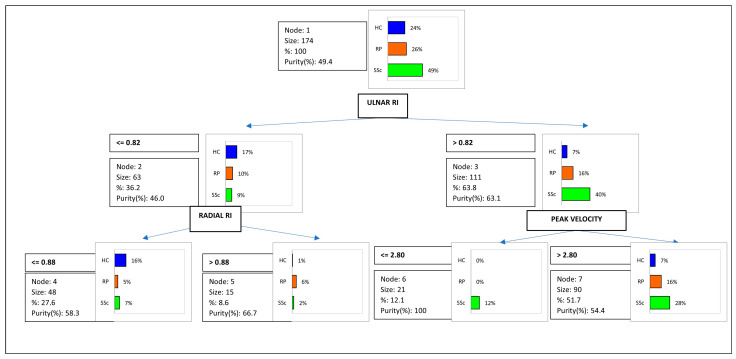
Decision tree: RI value leads to a more precise classification of Systemic sclerosis patients.

In the multivariate analysis, the PCA plot performed to distinguish the three groups SSc, RP and HC showed that they separated on the basis of ulnar and radial PV and RI. The first and second components explained 52.58% and 24.86% of the total variance. A decision tree model was applied to ulnar and radial PVs and RIs in SSc, RP and HC groups. The model classified 70% of SSc patients with ulnar RI > 0.82 and ulnar PV ≥ 2.8 cm/s. Cut-off values of ulnar RI ≤ 0.82 and radial RI < 0.88 were found for 94% of HCs (Figure 1). ROC curve results are reported in Table 3.

**Figure 2:**
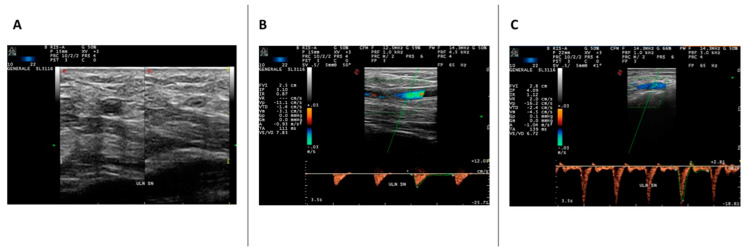
(**A**) Volar axial scan over left wrist, just proximal to Guyon tunnel, in a patient affected with SSc: 10–22 MHz liner probe. In the left side of image, the ulnar artery is visualized without compression; the intima media complex is thickened and mildly hyperechoic, and the lumen of the vessel shows echoic material (suspect for thrombotic material or slow flow). In the right side a compression is performed with the probe, and a not compressible thickened arterial wall is demonstrated. (**B**) Volar longitudinal scan over left wrist, just proximal to Guyon tunnel, in patient affected with SSc: 10-22 MHz liner probe. The CDUS demonstrates the abrupt reduction in flow into the distal ulnar artery, because of subtotal occlusion. The SWA before the occlusion shows a reduced flow (with low systolic PV of 11.1 cm/s) with substantial absence of flow in the diastolic phase with a large systolic phase, and high resistance. (**C**) Volar longitudinal scan over left wrist, just proximal to Guyon tunnel, in patient affected with primary Raynaud: 10–22 MHz liner probe. The CDUS-SWA demonstrates a triphasic flow into the ulnar artery with normal systolic PV and high resistive index.

In 12 patients (28%), we found UAOs (Figure 2). In nine cases (75%), UAOs were bilateral. A high prevalence of UAOs (42%) was found in SSc patients with the pattern “late” at NVC (*p* = 0.0264). The 12 SSc patients with UAOs had DUs in 10 cases (83.3%). On the contrary, among those patients showing DUs (21%), only 10 had UAOs (47.6%)

To stratify SSc patients according to the presence/absence of UAO, binary logistic regression analysis was performed in order to understand or predict the effect of clinical and US features (patterns in the capillaroscopy, disease duration, treatment, DU and radial and ulnar PV and RI) on UAO development. The goodness-of-fit statistics showed a Chi-squared associated with the Log ratio (L.R.) of 0.0001. From the probability associated with the Chi-squared tests, the Type II analysis showed that the variables that most influence the development of UAOs were DUs (value 3.39, OR 29.7, CI95% 4.9–31.6, *p* < 0.0001), ILD (value −2.91, OR 2.9, CI95% 0.5–3.3, *p* = 0.002) and patterns in the capillaroscopy (value 1.31, OR 3.72, CI95% 1.65–8.4, *p* = 0.002). From the statistical analysis, a positive significant correlation was shown between mRSS and ILD (*p* = 0.283 r = 0.033), between mRSS and DU (r = 0.344 *p* = 0.012) and between DU and ILD (r = 0.303 *p* = 0.024). No differences in terms of US results were found between the groups: limited vs. diffuse SSc or dividing the groups according to the antibodies. Male sex was associated with an increased frequency of UAOs (*p* = 0.042), with no difference in DU frequency between sexes. Male sex showed significance lower bilateral ulnar PV (*p* = 0.0010)

## 5. Discussion

In this study, peripheral vascular involvement of ulnar and radial arteries in SSc patients was assessed, showing that 28% of these patients had UAOs demonstrated at US evaluation. SSc patients with UAOs presented with DUs in 83.3% of the cases. Moreover, the RI at the ulnar artery was significantly higher in the SSc and RP groups than the HC group. US analysis led to the classification of 70% of SSc patients with an ulnar RI > 0.82 and ulnar PV> 2.8 cm/s. No UAOs were found in RP and HC groups. Interestingly, it emerged that radial arteries seem not to be involved in SSc vasculopathy. Therefore, our data suggest that peripheral macrovascular involvement is characteristic but not specific for SSc. Several studies confirmed that abnormalities of blood flow in digital and palmar arteries are already present at an early stage, while in a 5 years’ time follow-up, a RI > 0.70 seems to be a predictive marker of new DU development in SSc patients, together with NVC and a US vascular pattern of SSc arteries [18,19,20]. In our study, it was demonstrated that HCs also have an RI > 0.70, and this parameter should not be considered in order to explain or predict DU onset. On the contrary, we showed how cut-off values of ulnar RI ≤ 0.82 and radial RI < 0.88 classified 94% of HCs. However, it must be considered that the results of Colalillo et al. were found evaluating the digital arteries of the hand [19]. Baseline RI values of both radial and ulnar arteries can be comprised between 0.9–1, and the RI can modify according to the occlusion in one artery with the contralateral increasing its flow and decreasing the RI to guarantee blood flow to the hand [21]. Moreover, blood flow to the hand can be guaranteed by the complex and anastomotic vascularization of the hand, and this could explain why only some patients with UAOs have DUs or digital ischemia [22]. In agreement with our results, Schioppo et al. did not find any correlation between the RI and vascular parameters assessed by PDUS and NVC, suggesting that micro- and macrovascular involvement in SSc patients are potentially different and not completely overlapping entities, as supposed also by Sharp et al. [23,24]. In addition to these findings, our results also showed a positive correlation between mRSS and the presence of ILD, mRSS and DU and DU and ILD. Most of these elements are recognized as negative prognostic factors and reported as markers of vasculopathy [4,25]. No correlation was found between the clinical negative prognostic factor and UAOs, with the exception of a higher frequency of UAOs in male patients and a higher prevalence of late NVC scleroderma pattern, which are known to be negative prognostic factors [5], while an association was found between UAOs and the presence of ILD. Moreover, no difference was found between the RI value and negative prognostic factors. This finding suggests that peripheral vascular damage, assessed only by RI evaluation, could not be associated to severe visceral involvement. Our findings confirm some reports which considered UAO as a severity marker of vasculopathy even if we cannot maintain that DUs are fully caused by UAO [4,5,26,27].

No correlation was found between treatment or disease duration and UAO or US vascular parameters. Lescoat et al. demonstrated a correlation between UAO and many clinical parameters which were not showed in our paper. Particularly, the prevalence of UAO in Lescoat’s cohort was higher than in ours, with 37% of monolateral UAO and 24% of bilateral UAO [5]. This difference could be explained by the few numbers of enrolled patients and a higher disease activity of Lescoat’s cohort. However, to our knowledge, no other papers tried to assess differences in three different groups. On the other side, some papers assessed how to distinguish primary and secondary RP at US assessing the RI, flow and volume of digital arteries using a cold-stimulation test [16,20]. Our result cannot permit to differentiate SSc-RP from primary RP only by RI results. Specificity and the prognostic value of vascular involvement along with immune response which lead to the consequent fibrotic process are yet unclarified in SSc and RP, and this fibrotic process is a typical feature of SSc and a common sign of many CTDs that cannot be easily distinguished on a clinical level from primary RP, where vasospasm is not associated with a pathological condition [21]. However, US could be useful to differentiate HCs from RP patients, whether related or not to SSc, considering that the RI is lower in HCs. Luders et al. showed that the CDUS of hand and finger arteries allows to quantity the extension of SSc vasculopathy and signs of chronic bad perfusion, where patients with vascular damage showed higher percentage of DUs [25]. Moreover, the same authors recently proposed an algorithm to predict the development of new DUs in SSc using US vascular evaluation, requiring 45 min [23]. Our US approach is certainly less invasive and faster, requiring only a few minutes of US evaluation (around 10 min for both wrists) of ulnar and radial arteries. Finally, there is still the unmet need to understand the reason of UAO rather than other vessels. In this regard, UAO could be caused by the endothelial damage known in the pathogenesis of SSc rather than atherosclerosis. Indeed, the absence of correlation of dyslipidemia, hypertension and smoke with UAOs of SSc patients was demonstrated, also in accord with our findings [5]. Unfortunately, we did not evaluate the history of other cardiovascular risk factors such as dyslipidemia or hyperglycemia, considering that diabetes could be more responsible for micro-angiopathy rather than macrovascular damage [28]. Moreover, even if it was not our aim, we did not find any clear sign of atheroma at US ulnar artery evaluation. On the other hand, an association between anti-endothelin autoantibodies and UAO was not found [29]. Furthermore, dated papers have shown how ulnar artery specimens of SSc patients who underwent wrist revascularization presented a marked thickening and intimal fibrosis [6].

Our paper presents several limitations. We have to consider the operator dependency of US results and the limited number of enrolled patients and the disease activity, evaluated only by mRSS, which may have an influence on US results in terms of UAO together with the absence of other CTD group controls. Furthermore, the RP group was younger than the SSc group, while it was very similar in terms of age to the HC group, showing against this last group a significant difference in terms of RI, so we suppose that this difference would not drastically change our findings.

We have no data about patients with a very early onset of SSc or prospective evolution of DU according to the vasculopathy. Furthermore, it must be considered that a large number of patients were treated with vasodilators (calcium channel blocker, ERA, iloprost and sildenafil) or acetylsalicylic acid (ASA), considering the mean disease duration of 11 years. This could represent a limitation because it is not possible to know the real prevalence of UAO without these drugs or even the real prevalence of pulmonary artery hypertension; we could speculate that the vasodilator effect of the treatment could protect patients from UAO, but we did not demonstrate this result neither could we say that vasodilators are protective against UAO. However, a recent paper from the EUSTAR group was published underling how a low dose of ASA could be protective against DUs [30].

## 6. Conclusions

UAO is a typical feature of SSc, present in 28% of the cases, with 75% of those bilateral. UAO is associated with ILD and late NVC pattern. DUs are very frequent in cases of UAO (83%). UAO could be the expression of the macrovascular involvement, potentially linked to the presence of systemic fibrosis. The RI is a not specific index for SSc vasculopathy considering its high value in patients with primary RP. In this context, US could represent a simple and useful tool for evaluating patients at high risk of DU development and to identify vascular abnormalities associated with SSc.

## Figures and Tables

**Table 1:** Features of the evaluated population. SSc: systemic sclerosis; RP: Raynaud phenomenon; HC: healthy controls; BMI: body mass index; UAO: ulnar artery occlusion; ILD: interstitial lung disease; mRSS: modified Rodnan skin score; DLCO: diffusion lung carbon oxide; PAPs: pulmonary artery pressure; ERA: endothelin receptor antagonist; MMF: mycophenolate mofetil; MTX: methotrexate; AZA: azathioprine; RTX: rituximab; NINT: nintedanib; CS: corticosteroids; ANA: antinuclear antibodies; ACA: anticentromere antibodies; ENA: extractable nuclear antigens; np: not performed.

Parameters	SSc (n = 43)	RP (n = 23)	HC (n = 22)
Clinical findings			
Sex	F: 37 (86%) M: 7 (14%)	F: 16 (70%) M: 7 (30%)	F: 14 (63%) M: 8 (22%)
Mean age ± SD	60.5 ± 15	42.6 ± 15	44.5 ± 15
Smoking history	27 (62%)	5 (21.7%)	4 (18%)
Disease duration (years)	11.7 ± 10.2	0	0
BMI ± SD	24.2 ± 4.2	23.5 ± 3.3	23.5 ± 3.3
High blood pressure	17 (39.5%)	5 (21%)	2 (11%)
Disease subtype	28 (65%) limited 15 (35%) diffuse	0	0
Digital ulcer	21 (48%)	0	0
UAO	12/43 (28%) 9/12 bilateral	0	0
ILD	18 (65%)	0	0
mRSS	5.53 ± 6.80	0	0
DLCO% ± SD	74.7 ± 22	np	np
PAPs mmHg ± SD	28.5 ± 6.88	np	np
Capillaroscopic pattern			
Non-specific	5 (11.6%)	100%	np
Early	15 (34.8%)	0	np
Active	15 (34.8%)	0	np
Late	6 (14%)	0	np
Treatment			
Antiplatelets therapy	11 (25.5%)	1 (4.3%)	4 (18%)
Calcium channel blockers	9 (20.9%)	2 (8.7%)	0
Iloprost	14 (32.5%)	0	0
ERA	18 (42%)	0	0
Phosphodiesterase inhibitors	4 (9.3%)	0	0
Immunosuppressant (MMF, MTX, AZA and RTX)	17 (39%)	0	0
NINT	1 (2.3%)	0	0
CS	14 (32.5%)	0	0
Auto-Antibodies			
ANA	100%	0	np
ACA	20 (46.5%)	0	np
Anti-Scl70	13 (34.8%)	0	np
PM/Scl	2 (4.6%)	0	np
No ENA autoantibodies	8 (18.6%)	0	np

**Table 2:** Results of ultrasound evaluation. All data were expressed as mean ± standard deviation. SSc: systemic sclerosis; RP; Raynaud phenomenon; HC: healthy controls; PV: peak velocity; RI: resistance index.

Patients	Radial PV cm/s	Radial RI	Ulnar PV cm/s	Ulnar RI
SSc	24.5 ± 11.7	1.02 ± 0.198	16.1 ± 12.9	1.00 ± 0.209
RP	26.2 ± 20.4	1.00 ± 0.212	20.9 ± 11.9	0.964 ± 0.233
HC	30.9 ± 11.9	0.844 ± 0.180	26.6 ± 10	0.821 ± 0.216

**Table 3:** Results of ROC curve analysis discriminating patients (SSc and RP) and controls (HC) according to radial PV and RI and ulnar PV and RI. SSc: systemic sclerosis; RP; Raynaud phenomenon; HC: healthy control; PV: peak velocity; RI: resistance index; AUC: area under the curve.

	SSc vs. HC				RP vs. HC			
	Radial PV	Radial RI	Ulnar PV	Ulnar RI	Radial PV	Radial RI	Ulnar PV	Ulnar RI
AUC (%)	67.8	75.7	73.6	74.9	67.5	69.8	66.9	68.9
*p* value	0.001	<0.001	<0.001	<0.001	0.0049	0.0015	0.0062	0.0023
Cut-off value	25.25	0.85	22.2	0.82	24.85	0.83	22.2	0.82
Specificity (%)	61.9	71.4	66.7	69	66.7	66.7	66.7	69
Sensitivity (%)	61.6	73.3	67.4	75.4	66.7	73.3	60.9	60.9

## Data Availability

Data available on request due to restrictions for privacy.
